# Regulation of kisspeptin and gonadotropin-releasing hormone expression in rat placenta: study using primary cultures of rat placental cells

**DOI:** 10.1186/s12958-015-0083-3

**Published:** 2015-08-13

**Authors:** Aki Oride, Haruhiko Kanasaki, Tselmeg Mijiddorj, Unurjargal Sukhbaatar, Tomoko Ishihara, Satoru Kyo

**Affiliations:** Department of Obstetrics and Gynecology, Shimane University School of Medicine, 89-1 Enya-cho, Izumo, Shimane 693-8501 Japan

## Abstract

**Background:**

Gonadotropin-releasing hormone (GnRH) and kisspeptin in the hypothalamus are thought to be crucial components of the hypothalamic-pituitary-gonadal (HPG) axis and maintain reproductive function. These neuropeptides are also expressed in the placenta, where they may contribute to placental physiology. In this study, we examined how these peptides are regulated within the placenta.

**Methods:**

We used primary cultures of placental tissue from rats of 16–18 days gestation. After stimulation with estradiol, GnRH, kisspeptin, and neurokinin B (NKB), changes in placental GnRH, kisspeptin, and human chorionic gonadotropin (hCG) mRNA expression were evaluated by real-time quantitative RT-PCR analysis.

**Results:**

Immunocytochemical analysis showed that rat placental cells contained cells expressing kisspeptin or GnRH. GnRH and kisspeptin mRNA expression was significantly increased in placental cells in the presence of estradiol; NKB mRNA expression was also stimulated by estradiol. Stimulation of the cells with kisspeptin failed to stimulate GnRH mRNA expression. Conversely, both GnRH itself and NKB increased GnRH mRNA expression. Kisspeptin mRNA expression was not increased by kisspeptin itself; however, GnRH and NKB significantly increased kisspeptin mRNA expression. hCG expression was increased in the presence of estradiol. In addition, kisspeptin, GnRH, and NKB could stimulate the expression of hCG mRNA in placental cells.

**Conclusions:**

Our experiments using primary cultures of rat placental cells showed that GnRH, kisspeptin, and NKB expression was enhanced by estradiol, and unlike in the hypothalamus, kisspeptin did not control the expression of GnRH in placental cells. NKB might be located upstream of kisspeptin and GnRH, and these neuropeptides might be involved in the induction of hCG expression in placental cells.

## Background

After the discovery of inactivating mutations in the kisspeptin receptor of patients with idiopathic hypogonadotropic hypogonadism [1, 2], hypothalamic kisspeptin is now defined to be located upstream of gonadotropin-releasing hormone (GnRH)-expressing neurons and accepted as a crucial regulator of the hypothalamic-pituitary-gonadal (HPG) axis. Kisspeptin is a powerful stimulator of GnRH, and GnRH induces the secretion of gonadotropin from the anterior pituitary and maintains reproductive function by stimulating follicular growth and the synthesis of sex steroids [3].

GnRH and kisspeptin are expressed not only in hypothalamic neurons but also in a number of peripheral organs. Extrahypothalamic GnRH and its receptors are present in various tissues including the ovary, testis, prostate, and mammary gland [4]. The existence of a GnRH-like peptide in human placenta was first reported by Gibbons et al. and it was found to be identical to GnRH in the hypothalamus [5]. A receptor for GnRH was also identified in human placenta [6]. GnRH has been detected in extravillous cytotrophoblasts in human placenta during the 1st trimester [7], and the existence of GnRH receptors in these cells was also reported [8]. GnRH in placental tissues has been suggested to regulate the invasive differentiation of primary extravillous cytotrophoblasts by modulating the matrix metalloprotease or plasminogen system [9, 10]. Placental GnRH is also suggested to be involved in the autocrine/paracrine regulation of chorionic gonadotropin biosynthesis [11, 12].

The expression of kisspeptin and its receptor has also been demonstrated in a variety of tissues; kisspeptin is mainly expressed in the placenta, with much lower expression levels in the liver, pancreas, small intestine, testis, and brain [13]. Kisspeptin and its receptors are expressed strongly in the placenta, and this observation is paralleled by the finding that the plasma levels of kisspeptin are high in pregnant women [14]. In human placenta, kisspeptin mRNA is expressed in the syncytiotrophoblast, which develops from the fusion of cytotrophoblasts and represents the placental interface with the maternal circulatory system. The maternal decidua is devoid of kisspeptin. Kisspeptin receptors are expressed in the syncytiotrophoblast, villous, and invading extravillous trophoblasts [15]. Thus, kisspeptin and its receptor signaling have been identified as inhibitory regulators of trophoblast invasion. It was reported that the synthesis of kisspeptin inhibited extravillous cytotrophoblast migration without affecting their proliferation [15].

The physiological importance of GnRH and kisspeptin signaling are well understood as positive regulators of the HPG axis, in which GnRH is under the control of kisspeptin. Conversely, it unknown whether there is any relationship between the expression of GnRH and kisspeptin in the placenta. In this study, we used primary cultures of rat placental cells and examined how these neuropeptides are regulated.

## Materials and methods

### Materials

The following chemicals and reagents were obtained from the indicated sources: fetal bovine serum (FBS) (GIBCO, Invitrogen, Carlsbad, CA); Dulbecco’s modified Eagle’s medium (DMEM), water soluble β-estradiol (E2), GnRH, and penicillin-streptomycin (Sigma-Aldrich Co., St. Louis, MO); kisspeptin (Kp-10) (ANA SPEC, Fremont, CA); neurokinin B (NKB) (Sigma-Aldrich Co., St. Louis, MO); and DNase I (Promega Co., Madison, WI).

### Placental cell culture

Placental tissue was processed to obtain trophoblast primary cultures as described previously [16, 17]. Briefly, term placental tissue obtained from rats of 16–18 days gestation was cut into small pieces and digested for 30 min with 0.25 % trypsin and DNase I (300 U/mL) at 37 °C with gentle agitation. Then, the suspension was filtered through a cell strainer with 100-μm pores to remove undigested material. For washing, the supernatant was collected and resuspended in DMEM and centrifuged at 500 × *g* for 5 min, and the pelleted cells were washed once more. Finally, the suspension was layered over a Percoll solution (Percoll gradient was made from 70–5 % Percoll in 5 % steps of 2 ml each by dilution of 90 % Percoll with Hank’s balanced salt solution) and centrifuged at 1000 × *g* for 20 min. The middle layer was removed and washed with DMEM. Pennington et al. confirmed a relatively pure trophoblast cell population could be obtained from placental tissue using this protocol [16]. The isolated trophoblast cells were cultured in high-glucose DMEM containing 10 % heat-inactivated FBS and 1 % penicillin-streptomycin at 37 °C in a humidified atmosphere of 5 % CO_2_ in air. The protocol was approved by the committee of the Experimental Animal Center for Integrated Research at Shimane University.

### Immunocytochemistry

The cells were fixed on coverslips by 10 min methanol treatment at −20 °C. After 10 min of dehydration at 25 °C, the cells were treated with 0.2 % Triton X-100 in phosphate-buffered saline for permeabilization. Nonspecific antibody binding was blocked by preincubation with 1 % albumin, followed by an overnight incubation at 4 °C with an anti-kisspeptin antibody (1:500 dilution) (Millipore, Billerica, MA) and anti-GnRH 1 antibody (1:50 dilution) (Santa Cruz Biotechnology, Inc., Santa Cruz, CA). To visualize kisspeptin and GnRH, the cells were stained with secondary antibodies according to the manufacturer’s instructions for the use of a Histofine SAB-PO (MULTI) Kit (Nichirei Bioscience, Inc., Tokyo, Japan). The biotinylated secondary antibodies were coupled to streptavidin-biotinylated horseradish peroxidase and the reaction was visualized using diaminobenzidine as a chromogen. Chemiluminescence images of the cells were obtained with a microscope (Olympus BX41; Olympus, Tokyo, Japan).

### RNA preparation, reverse transcription, and real-time quantitative reverse transcription-polymerase chain reaction

Total RNA from placental cells was extracted using TRIzol-S (Invitrogen Life Technologies, Carlsbad, CA) according to the manufacturer’s instructions. To obtain cDNA, 1.0 μg total RNA was reverse transcribed using an oligo-dT primer (Promega, Madison, WI) and prepared using a First-Strand cDNA Synthesis Kit (Invitrogen Life Technologies, Carlsbad, CA) in reverse transcription (RT) buffer. The preparation was supplemented with 10 mM dithiothreitol, 1 mM of each dNTP, and 200 U RNase inhibitor/human placenta ribonuclease inhibitor (Code No. 2310; Takara, Tokyo, Japan) in a final volume of 10 μL. The reaction was incubated at 37 °C for 60 min. Quantification of GnRH, kisspeptin, and human chorionic gonadotropin (hCG) mRNA was obtained through real-time quantitative polymerase chain reaction (PCR; ABI Prism 7000; Perkin Elmer Applied Biosystems, Foster City, CA) following the manufacturer’s protocol (User Bulletin No. 2) and utilizing a Universal Probe Library Probe and Fast Start Master Mix (Roche Diagnostics, Mannheim, Germany). Using specific primers for GnRH (forward: 5′-ACTGTGTGTTTGGAAGGCTGC-3′ and reverse: 5′-TTCCAGAGCTCCTCGCAGATC-3′), kisspeptin (forward: 5′-ATGATCTCGCTGGCTTCTTGG-3′ and reverse: 5′-GGTTCACCACAGGTGCCATTTT-3′), NKB (forward: 5′-CCAGTGTGTGAGGGGAGCA-3′ and reverse: 5′-TCCAGAGATGAGTGGCTTTTGA-3′), and hCG (forward: 5′-ACATGGGCATCCAAGGAGCCGCTT-3′ and reverse: 5′-CGCACATCGCGGTAGTTGCACA-3′), the simultaneous measurement of mRNA and GAPDH permitted normalization of the amount of cDNA added per sample. For each set of primers, a no template control was included. Thermal cycling conditions were as follows: 10 min denaturation at 95 °C, followed by 40 cycles of 95 °C for 15 s and 60 °C for 1 min. The crossing threshold was determined using PRISM 7000 software, and postamplification data were analyzed using the delta-delta CT method in Microsoft Excel.

### Statistical analysis

All experiments were repeated independently at least three times, and each experiment was performed in duplicate for each experimental group. Data are expressed as the mean ± standard error of the mean (SEM). Statistical analysis was performed using one-way analysis of variance, followed by Newman-Keuls multiple comparison test.

## Results

### GnRH and kisspeptin expression in rat placental cells

**Fig. 1:**
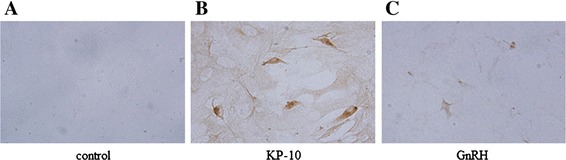
Staining of rat placental cells for GnRH and kisspeptin. Cultures of placental cells from rats at 18 weeks of gestation were immunostained using anti-kisspeptin (Kp-10) (**b**) and anti-GnRH (**c**) antibodies. The negative control is shown in (**a**) (control). The colors were developed by streptavidin-biotinylated horseradish peroxidase. Magnification, ×20

Primary cultures of rat placental cells collected from rats of 16–18 days gestation were cultured. The cultured placental cells contained several types of cells, but the majority of cells were considered to be trophoblasts according to the method used for cell collection. Immunostaining showed the expression of GnRH and kisspeptin in rat placental cells (Fig. 1).

### Effect of estradiol on GnRH, kisspeptin, and NKB mRNA expression

**Fig. 2:**
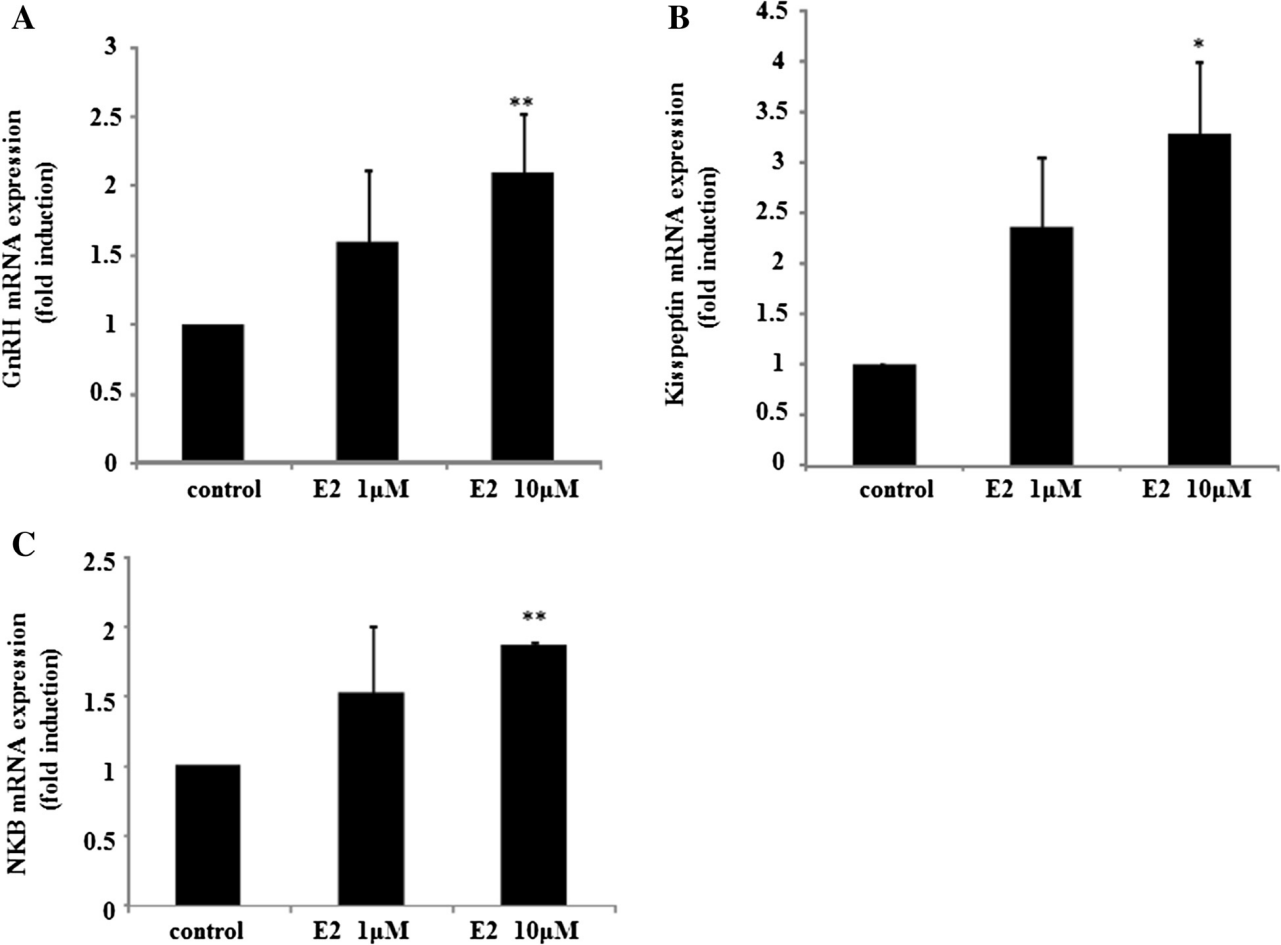
Effect of estradiol on GnRH, kisspeptin, and NKB expression in placental cells. Primary cultures of rat placental cells were cultured in the presence or absence (control) of 1 μM or 10 μM estradiol (E2) for 24 h. Then, GnRH (**a**), kisspeptin (**b**), and NKB (**c**) mRNA levels were measured by quantitative real-time PCR after mRNA extraction and reverse transcription. Samples for each experimental group were run in duplicate and normalized to GAPDH mRNA levels as a housekeeping gene. Results are expressed as fold stimulation over the unstimulated group/control. Values are the mean ± SEM of fold stimulation taken from independent experiments. **P < 0.01, *P < 0.05 vs. control

We examined whether GnRH and kisspeptin, which are expressed in placental cells, were regulated by estradiol. By treatment with 10 μM estradiol for 24 h, GnRH mRNA expression was significantly increased by 2.08 ± 0.43-fold compared to non-stimulated cells. One micromolar estradiol slightly increased GnRH mRNA levels, but not significantly so (Fig. 2a). Similarly, 10 μM estradiol, but not 1 μM, significantly increased kisspeptin mRNA expression by 3.30 ± 0.70-fold compared to non-stimulated cells (Fig. 2b). In this series of experiment, we also examined NKB expression, which is coexpressed in kisspeptin-producing neurons in the arcuate nucleus (ARC) of the rat hypothalamus and positively regulates their activity [18, 19]. NKB expression was also stimulated in the presence of 10 μM estradiol by 1.85 ± 0.03-fold compared to non-stimulated cells (Fig. 2c).

### Effect of kisspeptin on GnRH mRNA expression

**Fig. 3:**
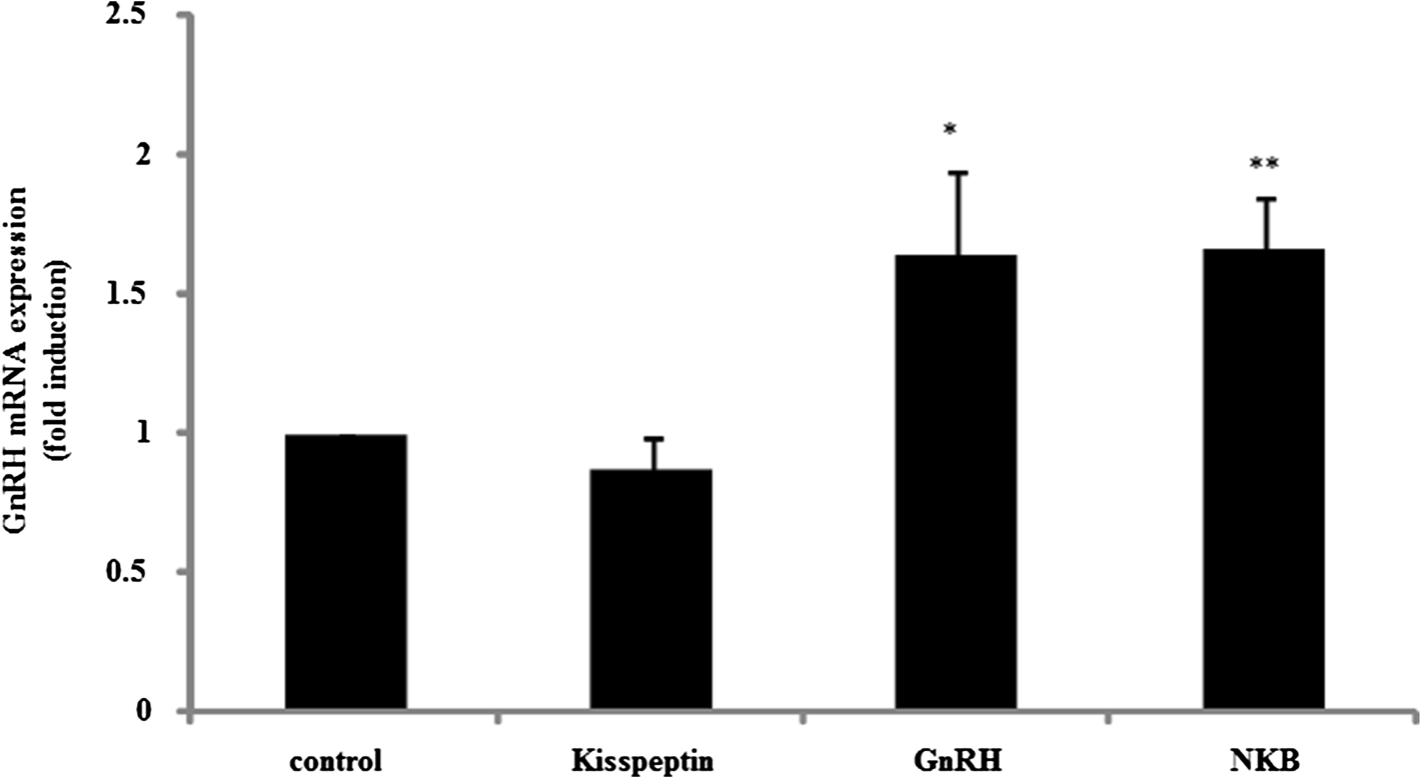
Effect of kisspeptin, GnRH, and NKB on GnRH mRNA expression in placental cells. Primary cultures of rat placental cells were treated with kisspeptin (100 nM), GnRH (100 nM), or NKB (100 nM) for 24 h. Then, GnRH mRNA levels were measured by quantitative real-time PCR after mRNA extraction and reverse transcription. Samples for each experimental group were run in duplicate and normalized to GAPDH mRNA levels as a housekeeping gene. Results are expressed as fold stimulation over the unstimulated group/control. Values are the mean ± SEM of fold stimulation taken from independent experiments. **P < 0.01, *P < 0.05 vs. control

Increasing evidence supports the hypothesis that kisspeptin controls gonadotropin secretion by stimulating GnRH-positive neurons in the hypothalamus. Next, we examined the effect of kisspeptin on GnRH-producing placental cells. Kisspeptin failed to stimulate GnRH mRNA expression in placental cells. GnRH itself slightly increased GnRH mRNA expression by 1.64 ± 0.3-fold in these cells compared to non-stimulated cells. In addition, NKB slightly, but significantly, increased GnRH mRNA expression by 1.67 ± 0.18-fold compared to non-stimulated cells (Fig. 3).

### Kisspeptin expression is increased by stimulation with GnRH and NKB

**Fig. 4:**
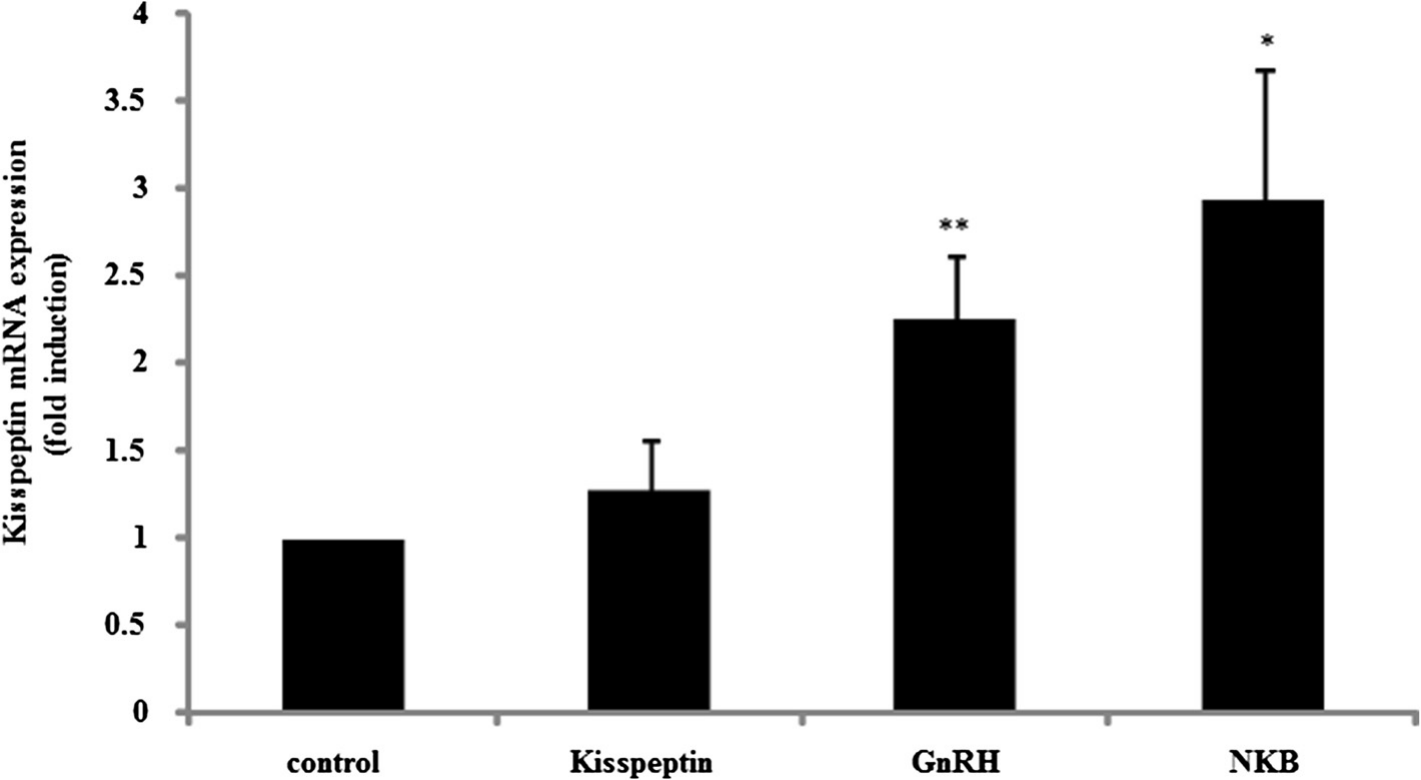
Effect of kisspeptin, GnRH, and NKB on kisspeptin mRNA expression in placental cells. Primary cultures of rat placental cells were treated with kisspeptin (100 nM), GnRH (100 nM), or NKB (100 nM) for 24 h. Then, kisspeptin mRNA levels were measured by quantitative real-time PCR after mRNA extraction and reverse transcription. Samples for each experimental group were run in duplicate and normalized to GAPDH mRNA levels as a housekeeping gene. Results are expressed as fold stimulation over the unstimulated group/control. Values are the mean ± SEM of fold stimulation taken from independent experiments. **P < 0.01, *P < 0.05 vs. control

Next, we examined how kisspeptin mRNA is regulated in placental cells. Kisspeptin itself did not modify its own gene expression. On the contrary, GnRH significantly increased kisspeptin mRNA expression by 2.27 ± 0.36-fold compared to non-stimulated cells. NKB also stimulated kisspeptin expression in placental cells by 2.95 ± 0.75–fold compared to non-stimulated cells (Fig. 4).

### hCG gene expression is enhanced by kisspeptin, GnRH, and NKB

**Fig. 5:**
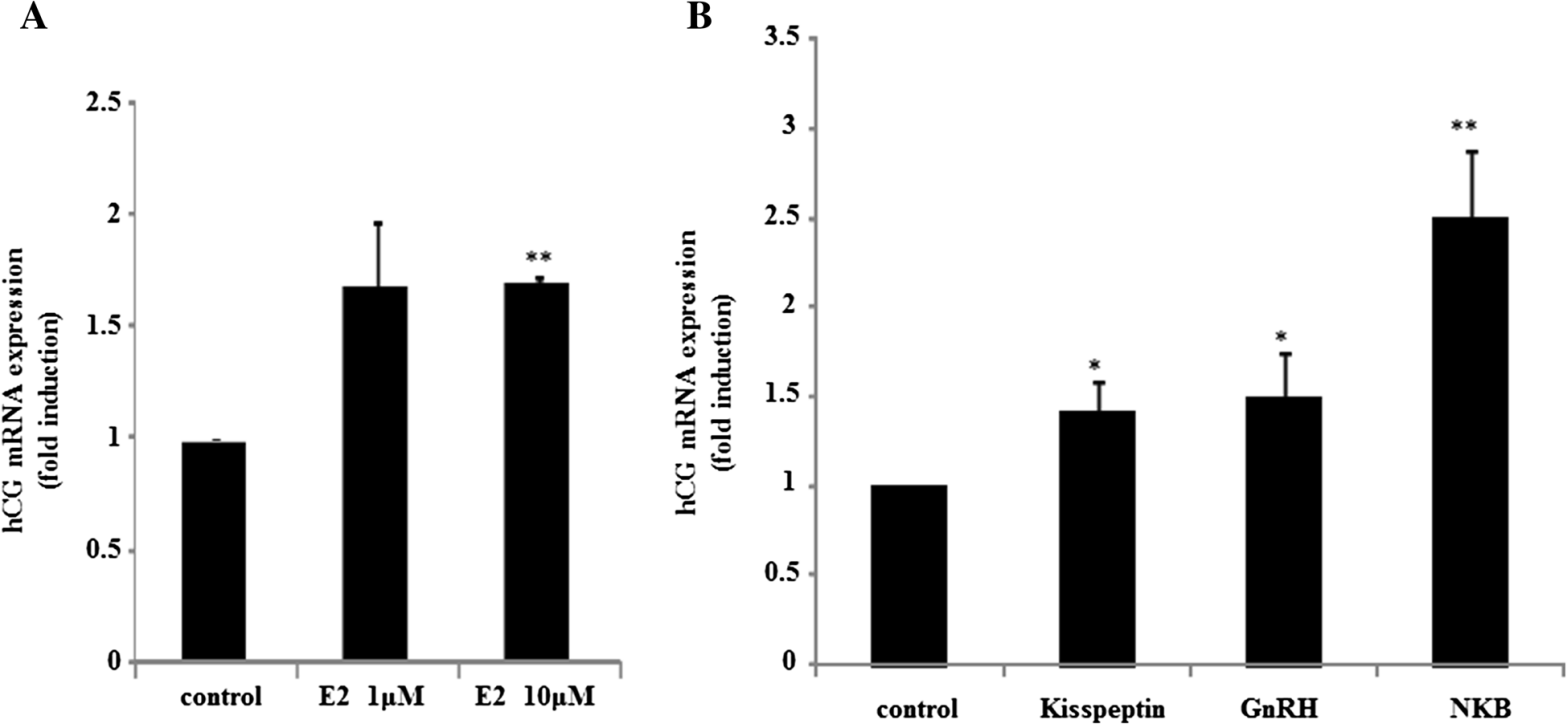
hCG expression by estradiol and neuropeptides. Primary cultures of rat placental cells were cultured in the presence or absence (control) of estradiol (E2) (**a**), kisspeptin (100 nM), GnRH (100 nM), or NKB (100 nM) (**b**) for 24 h. Then, hCG mRNA levels were measured by quantitative real-time PCR after mRNA extraction and reverse transcription. Samples for each experimental group were run in duplicate and normalized to GAPDH mRNA levels as a housekeeping gene. Results are expressed as fold stimulation over the unstimulated group/control. Values are the mean ± SEM of fold stimulation taken from independent experiments. **P < 0.01, *P < 0.05 vs. control

Finally, we examined the expression of hCG mRNA in placental cells. Before this experiment, we confirmed that hCG mRNA was amplified by RT-PCR using specific primers for human hCG in rat placental cells (data not shown). Similar to its effects on GnRH and kisspeptin, 10 μM estradiol significantly increased hCG mRNA expression in placental cells (Fig. 5a). Both kisspeptin and GnRH significantly stimulated hCG expression by 1.40 ± 0.17-fold and 1.49 ± 0.25-fold, respectively, compared to non-stimulated cells. In addition, NKB increased hCG mRNA expression by 2.51 ± 0.37-fold compared to non-stimulated cells, which was significantly higher than the effects of kisspeptin and GnRH (Fig. 5b).

## Discussion

Accumulating evidence supports the concept that the reproductive neuroendocrine axis is centrally regulated by inputs from kisspeptin-expressing neurons to GnRH-expressing neurons, which subsequently regulate the release of luteinizing hormone (LH) and follicle-stimulating hormone from the anterior pituitary. The direct and potent actions of hypothalamic kisspeptin at the level of GnRH-positive neurons have been documented [20, 21], and kisspeptin can reportedly increase GnRH mRNA expression in GnRH-secreting neuronal cells [22, 23].

In addition, estradiol is one of the most important regulators of the neuronal activity of kisspeptin and GnRH. Previous studies provided evidence that the estradiol-dependent induction of kisspeptin mRNA in the anteroventral periventricular nucleus (AVPV) may play a role in mediating the preovulatory GnRH/LH surge in female animals [24, 25]. Conversely, the expression levels of kisspeptin mRNA in the ARC increase upon gonadectomy and decrease upon steroid replacement in mice [26]. That is, estradiol, kisspeptin, and GnRH, which are present in the hypothalamus, affect each other and modulate their functions.

In addition, we examined the effect of NKB in the present study. Inactivating mutations in human genes encoding NKB (TAC3) and neurokinin 3 receptor cause idiopathic hypogonadotropic hypogonadism [27, 28], and low circulating levels of gonadotropins can be rescued by the administration of exogenous GnRH [29]. Kisspeptin neurons within the ARC are referred to as KNDy neurons because kisspeptin, NKB, and dynorphin are coexpressed in these neurons [18], and NKB has been suggested to act autosynaptically or transsynaptically to regulate kisspeptin secretion [30]. These observations suggest that NKB is located upstream of kisspeptin in the newly recognized HPG axis.

Previous reports demonstrated the presence of GnRH and kisspeptin in peripheral placental tissues [7, 15], but it remains unknown whether the GnRH expressed in placental cells is under the control of kisspeptin. To examine the physiological significance of extra-hypothalamic GnRH and kisspeptin, the current experiments were conducted. In primary cultures of rat placental cells, both kisspeptin- and GnRH-immunoreactive cells were detected using specific antibodies. According to the protocol used to obtain trophoblasts from the rat placenta [16, 17], the majority of these placental cells were considered to be trophoblasts.

First, we examined the effect of estradiol on GnRH and kisspeptin expression in placental cells, and found that the expression of both GnRH and kisspeptin in placental cells responded to and was increased by estradiol (Fig. 2a and b). A previous report demonstrated that GnRH mRNA expression was increased in hypothalamic GnRH-positive neurons of male chickens after castration, and this increase was prevented by treatment with estrogen [31]. In addition, an experiment using an immortalized GnRH-producing cell model demonstrated that estradiol treatment reduces GnRH mRNA expression at the single cell level [22]. These observations suggest the inhibitory control of GnRH by estradiol exists in the hypothalamus in an indirect or direct manner. As placental GnRH was upregulated by estradiol, the nature of GnRH-producing cells within the placenta might be distinct from hypothalamic GnRH-positive neurons. Whereas, although kisspeptin expression in hypothalamic kisspeptin-positive neurons is regulated by estrogen, the actions of estrogen largely differ according to the population of kisspeptin-expressing neurons examined. In rodents, kisspeptin expression in kisspeptin-positive neurons within the AVPV is up-regulated by treatment with estrogen [32]. Conversely, in kisspeptin-positive neurons within the ARC that co-express NKB and dynorphin (i.e., KNDy neurons), estrogen treatment decreases kisspeptin mRNA levels [24, 32]. As placental kisspeptin expression was upregulated by estradiol, kisspeptin-producing cells within the placenta might have similar characteristics to kisspeptin-expressing neurons within the AVPV in rodents. In addition, NKB expression in placental cells was also upregulated by estradiol (Fig. 2c). Considering the facts that KNDy neurons expressing NKB in the ARC were negatively regulated by estradiol [32] and NKB is a stimulator of kisspeptin release [30], NKB-expressing cells in the placenta might have distinct characteristics from those in the hypothalamus.

It is obvious that kisspeptin is a gatekeeper of reproduction owing to its central role in the HPG axis, and many data have led to the general concept that kisspeptin-positive neurons activate GnRH-positive neurons in the hypothalamus [33, 34]. Studies of the role of kisspeptin on GnRH-positive neurons have already focused on the cellular secretion of GnRH, which was determined indirectly by measuring LH release [35, 36]. In addition, GnRH gene expression is also reportedly regulated in response to kisspeptin in GnRH-producing cell models [22, 37]. In our primary cultures of rat placental cells, kisspeptin failed to stimulate GnRH expression, but GnRH stimulated the expression of kisspeptin (Figs. 3 and 4). These observations suggest that, unlike in the hypothalamus, kisspeptin does not govern GnRH expression in placental cells. Although it is unknown whether GnRH activates kisspeptin-expressing neurons in the hypothalamus, GnRH could interact with kisspeptin-positive cells and regulate kisspeptin expression in the placenta.

In addition, it was revealed that stimulation of placental cells with NKB significantly increased both GnRH and kisspeptin mRNA expression (Figs. 3 and 4). NKB is a 10-amino-acid peptide belonging to the family of tachykinin-related peptides, such as substance P and neurokinin A, and NKB has neurotransmitter and neuromodulatory activities [38]. NKB is also expressed in the placenta and is thought to be involved in parturition because placental NKB mRNA levels increase at preterm labor [39] and NKB levels increase in maternal blood throughout pregnancy and decrease rapidly after delivery [40]. In addition, the high expression of NKB mRNA in chorionic villous samples at 11 weeks of gestation could be a significant marker for pre-eclampsia [41]. Considering previous observations, our current results implied that the NKB produced within the placenta plays some role in placental formation and it functions in cooperation with GnRH and kisspeptin.

Although a stimulatory effect of estradiol on GnRH and kisspeptin expression was observed in placental cells, it also stimulated hCG expression in these cells (Fig. 5a). In addition, we found that kisspeptin and GnRH can also stimulate hCG expression in placental cells. Furthermore, NKB stimulated hCG mRNA expression to a greater degree than either kisspeptin or GnRH (Fig. 5b). Previous studies hypothesized that the GnRH produced within the placenta might be involved in the autocrine/paracrine regulation of hCG biosynthesis [11, 42]. After kisspeptin expression was identified in the placenta, placental kisspeptin was proposed to play a role in the regulation of placental formation, and was defined as an inhibitor of trophoblast invasion and proliferation [15, 43]. On the contrary, the serum levels of hCG reportedly peaked around 8 weeks of gestation and decreased progressively with the concomitant increase in the serum levels of kisspeptin [44]. At present, the detailed mechanism by which neuropeptides, such as kisspeptin, GnRH, and NKB, increase hCG synthesis and why they could increase hCG expression in placental cells remain unknown. However, considering the observation that the increase of hCG mRNA expression by GnRH and kisspeptin was not drastic, they might exert only a partial effect on hCG biosynthesis in the placenta. Further studies are required to examine the control of hCG expression by these neuropeptides in placental cells.

## Conclusions

**Fig. 6:**
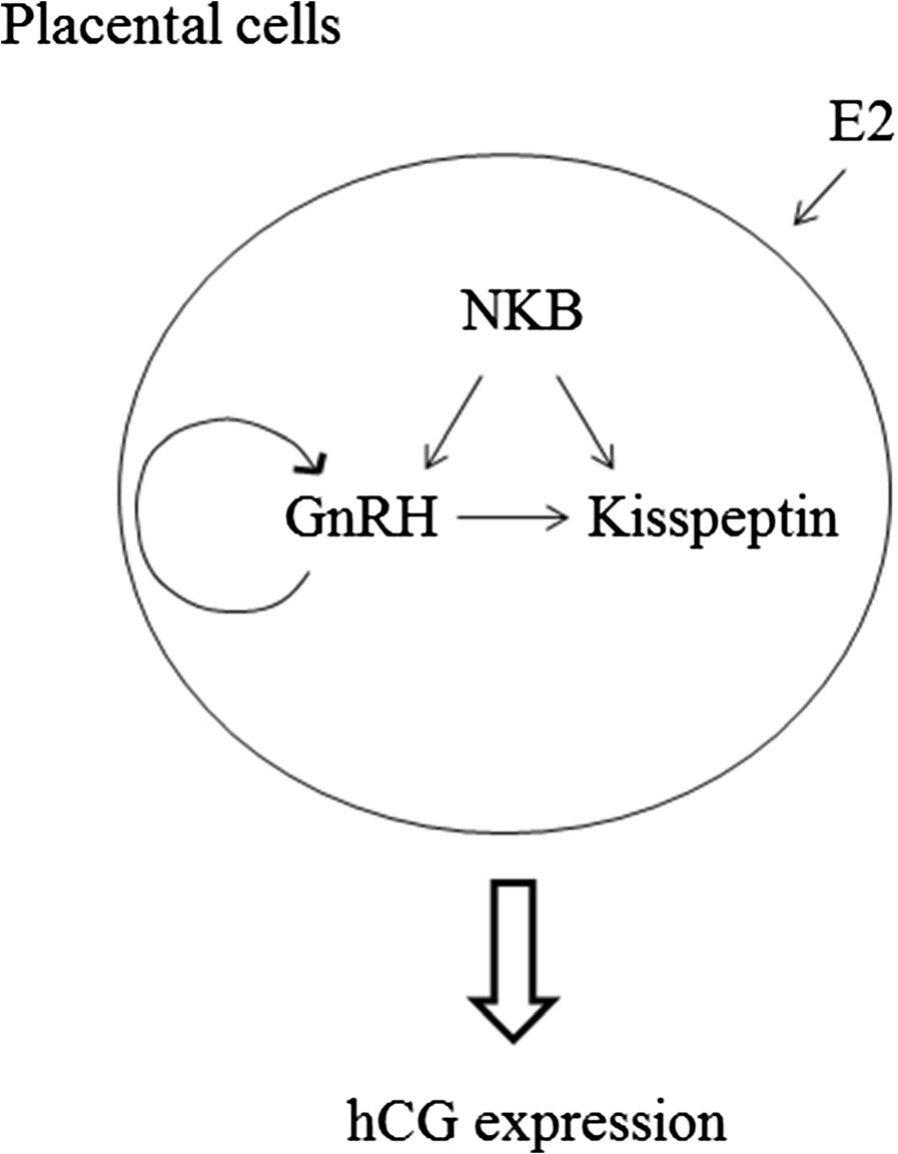
Schematic summary of the present study. Hypothalamic peptides, GnRH, kisspeptin, and NKB, were detected in primary cultures of placental cells. All of these peptides were up-regulated by estradiol (E2). GnRH expression was not regulated by kisspeptin, but was regulated by GnRH itself and NKB. Kisspeptin expression was up-regulated by GnRH and NKB. All of these neuropeptides increased hCG expression in placental cells

Our experiments using primary cultures of rat placental cells showed that GnRH, kisspeptin, and NKB expression was enhanced by estradiol, and unlike in the hypothalamus, kisspeptin did not control the expression of GnRH in placental cells. NKB might be located upstream of kisspeptin and GnRH, and these neuropeptides might be involved in the induction of hCG expression in placental cells. The interaction of kisspeptin, GnRH, and NKB and their function in placental cells are summarized in Fig. 6.

The axons of kisspeptin-positive neurons in the hypothalamus form dense capillary plexuses at the site of GnRH neurosecretion, and close contacts between kisspeptin-positive and GnRH-positive axons have been demonstrated [45]. As placental cells that produce GnRH, kisspeptin, and NKB are not neuronal cells, these neuropeptides are considered to interact in an autocrine/paracrine manner in the placenta. Neuropeptides produced within the placenta might work under different mechanisms of control to those in the hypothalamus. As it is evident that estrogens, such as estradiol and estrone, are produced within the placenta [12], we could speculate that the estrogens produced within the placenta might increase or maintain these peptide hormones by both autocrine and paracrine mechanisms and maintain hCG expression.
